# Prenatal Exposure to Perfluoroalkyl Substances and Behavioral Development in Children

**DOI:** 10.3390/ijerph13050511

**Published:** 2016-05-19

**Authors:** Ilona Quaak, Marijke de Cock, Michiel de Boer, Marja Lamoree, Pim Leonards, Margot van de Bor

**Affiliations:** 1 Department of Health and Life Sciences, Faculty of Earth and Life Sciences, Vrije Universiteit, De Boelelaan 1085, Amsterdam 1081 HV, The Netherlands; m.de.cock@vu.nl (M.d.C.); margot.vande.bor@vu.nl (M.v.d.B.); 2 Department of Health Sciences, Faculty of Earth and Life Sciences, Vrije Universiteit, De Boelelaan 1085, Amsterdam 1081 HV, The Netherlands; m.r.de.boer@vu.nl; 3 Institute for Environmental Studies, Faculty of Earth and Life Sciences, Vrije Universiteit, De Boelelaan 1085, Amsterdam 1081 HV, The Netherlands; marja.lamoree@vu.nl (M.L.); pim.leonards@vu.nl (P.L.)

**Keywords:** perfluoroalkyl substances, endocrine disrupting chemicals, Attention Deficit Hyperactivity Disorder, behavioral development, early life exposure, prenatal exposure, preschool children

## Abstract

***Background*:** In recent years, prevalence rates of behavioral disorders in children have increased. One factor possibly implied in the etiology of behavioral disorders is exposure to perfluoroalkyl substances (PFASs). The use of PFASs is highly integrated into everyday life, and exposure is ubiquitous. Exposure to PFASs during early life may be particularly harmful, as it represents a critical time window for brain development. However, research in the area is limited, especially among preschool children. The objective of the current study was to explore the relationship between prenatal exposure to several PFASs and behavioral development at the age of 18 months. ***Methods*:** Data from the Dutch cohort LINC (Linking Maternal Nutrition to Child Health) were used. Perfluorooctanesulfonic acid (PFOS) and perfluorooctanoic acid (PFOA) were measured in cord plasma. The total exposure of PFASs was also calculated (ΣPFASs). Behavioral development was assessed with the Child Behavior Checklist 1.5–5 (CBCL 1.5–5). The CBCL scales “Attention Deficit Hyperactivity Disorder” (ADHD) and “Externalizing problems” were used for further analysis. Separate regression models were composed for each combination, in which exposure levels were classified in tertiles. Both whole population and sex-stratified analyses were performed. A family history of ADHD, the educational level, smoking or using alcohol or illicit drugs during pregnancy were considered as confounders. In total, data from 76 mother-child pairs was included. ***Results*:** No significant associations were found between prenatal PFAS exposure and ADHD scores in the whole population and in the sex-stratified analyses. With regard to externalizing behavior, a significant negative association was found between the highest levels of ΣPFAS exposure and externalizing problem behavior in the whole population, but only in the crude model. After stratifying for sex, boys in the second and third tertile of exposure to PFOA presented significantly lower scores on the Externalizing Problem Scale than boys with the lowest exposure levels in the adjusted model. Girls exposed to higher levels of ΣPFAS exposure (T2) showed significantly lower scores on the Externalizing Problem Scale, in both crude and adjusted models. No significant associations with PFOS were found. ***Conclusions*:** Results from the current study show that prenatal exposure to PFOA was negatively related to externalizing behavior in boys. Results were different for boys and girls, emphasizing that mechanisms at work might be sex-dependent. However, results should be interpreted with caution as the sample size was small.

## 1. Introduction

In recent years, prevalence rates of behavioral disorders in children have increased [1]. In a recent population-based registry study from Germany, a general increase rate of 38.1% was reported for all mental disorders in children and adolescents between 2000 and 2007, with an increase rate of 70% for bipolar disorder, 18% for conduct disorder and over 200% for depressive disorder [2]. In Canada, an increase of antidepressant treatments was reported from 5.9 per 1000 children aged 0 to 19 in 1983, to 15.4 per 1000 in 2007 [3]. With regard to Attention Deficit Hyperactivity Disorder (ADHD), increasing prevalence rates were reported in the United States and Taiwan [4,5]. Hence, increasing prevalence rates of behavioral disorders among children appear to be a worldwide phenomenon, creating the need to gain more insight into their etiology. The increasing prevalence rates might be the result of more inclusive diagnostic criteria of the disorders, wider screening and more public awareness [6]. In addition, children are diagnosed at an increasingly younger age [6]. However, most authors believe that these factors alone do not sufficiently explain the rise in prevalence rates of disorders among children. There is consensus that most behavioral disorders are multi-factorial conditions, in which both genetic and environmental risk factors are involved in the etiology. Moreover, many behavioral disorders are heterogeneous: different symptoms can be observed among individuals with the same disorder, as is the case with ADHD and conduct disorder [7,8,9]. This heterogeneity emphasizes that different factors might be involved in the etiology.

One of these factors is early life exposure to chemicals. In the past decades, the production of manmade chemicals has increased [10]. Some of these compounds interfere with endocrine processes and are referred to as endocrine disrupting chemicals (EDCs). In the current study we will focus on the EDC class of perfluoroalkyl substances (PFASs), of which perfluorooctanic (PFOA) and perfluorooctanesulfonic acid (PFOS) are the most well known. PFASs have been widely used since the 1950s in, for example, cleaning products, paper and packaging, water-resistant and stain-resistant fabrics, leather and pesticides [11,12,13]. Since 2010, the use of PFOS has been restricted in the European Union, while the United States Environmental Protection Agency and the primary manufacturers of PFOA agreed on a gradual phase-out of PFOA by 2015 [14,15]. Human exposure mainly occurs through the transfer from food packages, the food chain and household dust [16]. Previous work has shown that PFOA and PFOS have been detected in serum, amniotic fluid, breastmilk and maternal and umbilical cord blood [17,18,19,20,21,22,23,24,25,26]. The ubiquitous presence and persistence of PFASs have led to an increased concern about the health effects of these compounds. Exposure has been related to adverse effects on physical health, including obesity, early menopause and changes in glucose and lipid homeostasis [27,28,29,30,31,32,33,34,35,36]. Health effects resulting from exposure to PFASs might differ, depending on the compound, and the timing and dose of the exposure. It has been demonstrated that the dose-response curve of some chemicals might not be linear, which underlines the potential of even very low doses of exposure to affect human health [37]. The developmental age at which exposure takes place is crucial in the understanding of the health effects [38]. As PFASs can pass through the placenta, exposure of the fetus starts early in gestation. The prenatal period is a particularly sensitive period for exposure to exogenous compounds as rapid structural and functional changes take place. Therefore, early life exposure to PFASs has the potential to affect fetal as well as child development [39]. 

Prenatal exposure of mice to PFOA has been related to changes in exploratory behavior in both male and females, while PFOS exposure has been related to decreased locomotion in male offspring [40]. In a human study by Stein *et al*., a negative relationship between *in utero* exposure to PFASs and ADHD at the age of 6 to 12 years was reported [25]. However, both positive and negative associations were reported when exposure was assessed at a later age [41,42,43]. Human studies assessing ADHD at younger ages are lacking.

The rapid increase in behavioral disorders, especially ADHD, among children has led to a need to further investigate their etiology. Given that exposure to PFASs is ubiquitous and has been related to various adverse health effects, it seems essential to further investigate a possible association with behavioral development, since human data are limited. The aim of the current study is to further explore the relationship between prenatal exposure to PFASs and behavioral development in children, in particular with regard to externalizing behavior. Externalizing behavior is defined as outward behavior whereby the child negatively reacts to the external environment [44,45,46]. Some descriptions of externalizing problem behavior include hyperactive, disruptive or aggressive behavior [47]. As some ADHD-associated behaviors are subsumed under externalizing behavior, this endpoint is also included. These relationships will be investigated for PFOS and PFOA individually and for ΣPFASs, representing the summation of the concentrations of PFOS, PFOA and five other perfluoroalkyl compounds. Based on the strong chemical similarities of the PFASs, we assume that these compounds may share a common pathway towards the exhibition of an adverse outcome such as specific behavioral disorders in children.

## 2. Materials and Methods 

### 2.1. Linking Maternal Nutrition to Child Health (LINC) Cohort

Data were used from the Dutch mother-child cohort LINC (Linking Maternal Nutrition to Child Health) [48,49]. The LINC cohort was set up in the region of Zwolle, a rural setting characterized by agriculture and stock-breeding. Participants were included between 2011 and 2013. All subjects gave their informed consent for inclusion before they participated in the study. Pregnant women were asked to participate during their first antenatal visit to the midwifery clinic. Exclusion criteria were pre-eclampsia, twin pregnancies and major congenital anomalies at birth. Women were eligible for participation if they were able to fill out Dutch questionnaires.

Participants were followed during pregnancy and after birth, until the children reached the age of 18 months. During this period, the women filled out questionnaires every three months on a variety of topics, including nutrition, lifestyle, mental wellness and indoor environment. The study was conducted in accordance with the Declaration of Helsinki, and the protocol was approved by the Medical Ethics Committee of the VU University Medical Centre (NL31941.029.10).

### 2.2. Chemical Exposure

Immediately after birth, umbilical cord blood was collected. Midwives and nurses collected as much blood as possible in EDTA tubes. Within twelve hours the blood was delivered to the hospital laboratory. The cord blood was then centrifuged at 2000 g for 10 min. The plasma layer (CP) was subsequently transferred to plasma tubes, which were stored at −80 °C.

Perfluorooctanoic acid (PFOA), perfluorooctanesulfonic acid (PFOS), perfluorohexane sulfonate (PFHxS), perfluoroheptane sulfonate (PFHpS), perfluorononanoic acid (PFNA), perfluoro-n-decanoic (PFDA), perfluoroundecanoic acid (PFUnDA) were determined and a sum variable was created (ΣPFASs). For the ΣPFASs, statistical analyses were performed expressing compounds in both nanogram per liter (ng/L) and in nanomolar (nM). Regarding the individual compounds, the focus will be on PFOA and PFOS, using wet weight values (expressed in ng/L), as the compounds are not lipophilic. The compounds were analyzed using a liquid chromatography-mass spectrometric (LC-MS) method, after solid phase extraction (SPE). All values were above the limit of quantification (LOQ), which was between 50 and 140 ng/L (0.12–0.34 nM) for PFOA, between 44 and 140 ng/L (0.09–0.28 nM) for PFOS, and between 165 and 404 ng/L (0.37–1.01 nM) for ΣPFASs (Table 1). 

#### Behavioral Assessment

At the age of 18 months parents filled out the Child Behavior Checklist 1.5–5 (CBCL) to assess the behavioral development of the child [50]. Parents had to indicate for 99 items on a three-point Likert scale whether a statement was very true (2 points), somewhat true (1 point) or not true (0 points) for the child. The scales “Externalizing Behavior” and “Attention Deficit Hyperactivity Problems” were used for the analysis. “Attention Deficit Hyperactivity Problems” is a DSM-based subscale consisting of six items; the minimum raw score is 0, the maximum raw score 12. The scale “Externalizing Problems” is composed of the empirically based scales “Attention Problems” and “Aggressive Behavior.” The minimum raw score is 0; the maximum raw score is 48.

For the analyses, raw scores were used. The calculation of the raw score was performed by the software program Assessment Data Manager (ADM), developed by Achenbach [50]. Raw scores are usually preferable for statistical analyses, as they reflect all differences among scores directly, without the effects of transformations [50]. In addition, differences at the lower end of the normal range are preserved. 

### 2.3. Covariates

Covariates were selected using literature [51,52,53,54,55,56]. Variables checked for confounding effects were family history, educational level, smoking, alcohol use and illicit drug use during pregnancy. The data were collected by means of questionnaires. Family history was assessed by asking parents whether any family members of the child were affected by ADHD (yes or no). Educational level was assessed by asking parents to indicate the highest educational level they had completed (for both parents separately) on an eight-point scale. The parents of the child were defined as having a high educational background if at least one of them had a bachelor’s or a master’s degree. Smoking, alcohol and illicit drug use were assessed by asking the mother if she had used any of these substances during the pregnancy. Information on covariate selection for the statistical models is given under “Analysis”. 

### 2.4. Analysis

Data analyses were comparable to previous studies performed on data from this cohort [48,49]. Linear regression analyses were carried out to examine the association between prenatal exposure to PFASs and behavioral development at the age of 18 months. As none of the independent variables showed a linear relationship with the outcomes of the CBCL used for the behavioral assessment, variables were split into tertiles, and analyzed as dummy variables. For each compound-outcome combination, a separate regression model was composed. The interaction term “sex” was added to the model, but was not significant in any of the models. However, sex-stratified analyses were performed as previous work has indicated that mechanisms of action of certain EDCs might be sex-dependent [48,49,57]. In addition, the power for interaction tests is reduced, especially when studying a small sample. Therefore, analyses were first performed for the entire study population, and subsequently stratified for sex. 

Crude and adjusted analyses were carried out for all regression models. A confounder was included in the analysis if there was a change of at least 10% in the regression coefficient of any of the exposure dummy variables after adding that covariate to the model. Illicit drug use could not be checked as a confounder as none of the participants used any drugs during pregnancy. 

Bias corrected accelerated confidence intervals were calculated based on 1000 bootstrap samples. The essence of the bootstrap method is to draw pseudo-samples (bootstraps) from the original sample itself [58]. Distributional assumptions are not required for bootstrapping; therefore, the method is suitable for nonlinear statistics or when the sample size is small, as was the case in the current study. In these cases, bootstrapping may provide more accurate confidence intervals than the standard approach. A confidence interval not crossing the null was considered to indicate statistical significance. Although we believe this was the most valid statistical approach, it should be noted that null-hypothesis tests based on *p*-values did not correspond to those based on the confidence intervals in all analyses. Finally, sensitivity analyses were carried out by redoing all analyses on data without outliers. Outliers were defined as data points more than 1.5 interquartile ranges above the third quartile, and more than 1.5 interquartile ranges below the first quartile. All analyses were carried out in SPSS version 21 (IBM SPSS Statistics for Windows, Armonk, NY, USA).

## 3. Results

### 3.1. Sample Characteristics

**Figure 1:**
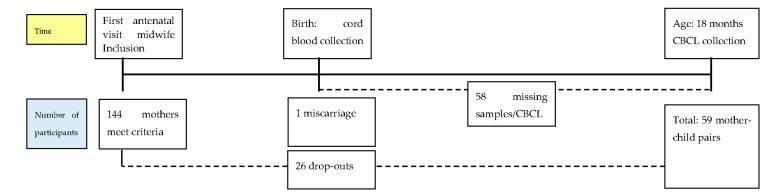
Flow chart of the number of participants and timeline of the study.

In Figure 1, a flow chart is presented of the number of participants throughout the study. A total of 144 mother-child pairs were initially included in the study. Twenty-six mother child-pairs dropped out of the study. One participant had a miscarriage. For 58 pairs, there were no CBCL and/or exposure data available. Therefore, the total number of included mother-child pairs was 59 (Figure 1).

In Table 2, an overview is provided of the characteristics for both boys (*n* = 34–38) and girls (*n* = 20–21). Educational level was high in both groups: more than 50% of the children had at least one parent with a bachelor’s or a master’s degree. With regard to ADHD, 16.7% of the boys had a family member affected by the disorder, compared to 10.0% of the girls. The mean ADHD score was 3.3 for boys, and 3.6 for girls. Externalizing scores were slightly higher in girls than in boys (9.6 *versus* 9.2).

### 3.2. Exposure Levels

In Table 1, exposure levels of the various compounds included are presented. The median PFOA level was 870.0 ng/L (2.10 nM), while the median PFOS level was 1600.0 ng/L (3.21 nM). The median of ΣPFASs was 2907.0 ng/L (6.24 nM). 

### 3.3. Associations between Perfluoroalkyl Substances (PFASs) and Behavioral Variables at 18 Months

#### 3.3.1. Attention Deficit Hyperactivity Disorder (ADHD) Symptoms

**Figure 2:**
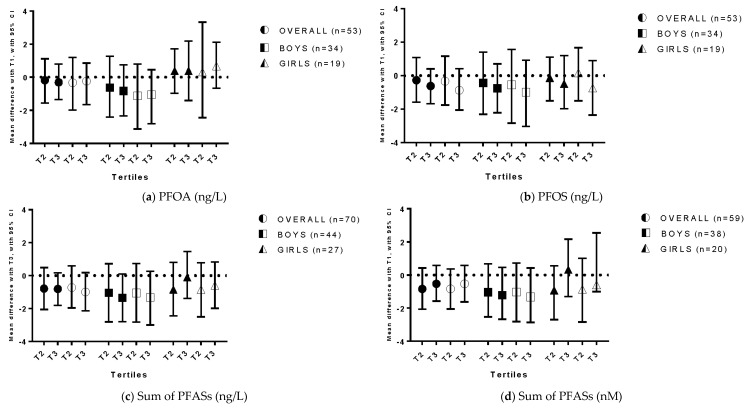
Exposure to perfluoroalkyl substances and scores on the Attention Deficit Hyperactivity Disorder Scale of the Child Behavior Checklist 1.5–5. (**a**) PFOA (ng/L); (**b**) PFOS (ng/L); (**c**) Sum of PFASs (ng/L); (**d**) Sum of PFASs (nM). The mean difference in ADHD scores between tertiles of exposure is reported, with T1 as a reference category. Filled symbols represent results from crude analyses, while empty symbols represent results from adjusted analyses.

No significant associations were found between prenatal exposure to PFASs and ADHD symptoms in the whole study population, nor in the sex-specific analyses (Figure 2 and Appendix Table A1, Table A2, Table A3 and Table A4). Analyses using ΣPFASs expressed in nM generated almost identical results to those using ng/L. In addition, only two children were categorized in different tertiles. 

#### 3.3.2. Externalizing Problems

**Figure 3:**
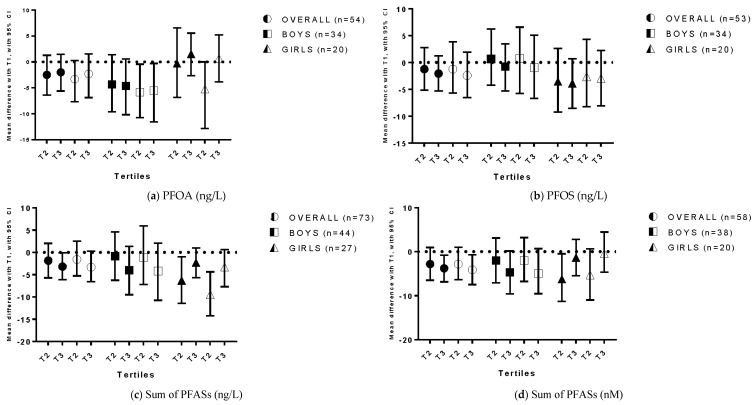
Exposure to perfluoroalkyl substances and scores on the Externalizing Behavior Scale of the Child Behavior Checklist 1.5–5. (**a**) PFOA (ng/L); (**b**) PFOS (ng/L); (**c**) Sum of PFASs (ng/L); (**d**) Sum of PFASs (nM). The mean difference in ADHD scores between tertiles of exposure is reported, with T1 as a reference category. Filled symbols represent results from crude analyses, while empty symbols represent results from adjusted analyses.

PFOA exposure was negatively related to the Externalizing Problem Scale in adjusted sex-stratified analyses (Figure 3a). Boys in the second and third tertile of exposure to PFOA presented lower scores on the Externalizing Problem Scale than boys with the lowest exposure levels (mean difference second tertile −5.87; 95% CI: −10.76, −0.43; mean difference third tertile −5.54; 95% CI: −11.57, −0.29). These associations were only found after adjusting for a family history of ADHD, smoking, alcohol use and education. No significant associations were found between PFOS exposure and scores on the Externalizing Problem Scale (Figure 3b). A significant negative association was also found between ΣPFAS exposure in ng/L and scores on the Externalizing Problem Scale, but only in crude analyses (mean difference T3 −3.19; 95% CI: −6.10; −0.07; Figure 3c). In the analyses using ΣPFASs expressed in nM results remained significant after adjustment for alcohol use during pregnancy (crude analyses mean difference T3 −3.80; 95% CI: −6.86; −0.79; adjusted analyses mean difference T3 −4.11; 95% CI: −7.49; −0.68; Figure 3c). After stratification for sex, a significant negative association was found for ΣPFAS exposure (ng/L) and externalizing problem behavior, but only in girls in the second tertile, in both crude and adjusted analyses (crude mean difference −6.30; 95% CI: −1.42; −0.99; adjusted mean difference −9.50; 95% CI: −14.21; −4.35; Figure 3c). In the analyses using ΣPFASs expressed in nM, the association was, however, not significant in the adjusted analyses (mean difference −5.29; 95% CI: −11.00; 0.67; Figure 3c). No significant associations were found between PFOS and externalizing problems, in the whole population nor in sex-stratified analyses.

### 3.4. Sensitivity Analyses

The sensitivity analyses excluding outliers showed similar results to the analyses on the full dataset. With respect to the null hypotheses tests, though, one difference was observed. The association between prenatal PFOA exposure in the second tertile and externalizing behavior in boys was no longer statistically significant (mean difference −6.03; CI: −10.66, −0.35). 

## 4. Discussion

The objective of the current study was to explore the relationship between exposure to PFASs, determined in cord blood, and behavioral development at the age of 18 months. A significant negative association was found between the highest levels of ΣPFAS exposure and externalizing problem behavior in the whole study population, but only in the crude model. After stratifying for sex, a significant negative association was found between prenatal PFOA exposure (T2 and T3) and externalizing behavior scores in boys, after adjustment for confounders. Girls exposed to higher levels of ΣPFAS exposure (T2) presented significantly lower scores on the Externalizing Problem Scale, in both crude and adjusted models. Analyses using ΣPFASs expressed in nM generated similar results to those using ng/L, except for a significant association between the third tertile of ΣPFASs and externalizing behavior in the adjusted models.

Exposure levels in the current study were rather low compared to levels from other birth cohorts [59,60,61]. The median PFOA level in the current study was 920 ng/L *versus* 3300 to 4100 ng/L in a recent Danish cohort using maternal blood [59]. Similarly, the median PFOS level varied between 20,400 and 22,400 ng/L, while in the current study, the median PFOS level was 1650 ng/L (Table 1). A recent Spanish study indicated that PFAS values in maternal serum and cord blood are positively correlated [62]. However, maternal serum values were typically higher than those found in cord blood. In a Korean cohort study, higher median PFOS and PFOA levels were found. 

Although the sample size of the current study was small and exposure levels were low, we did find significant results, emphasizing the potential effect of low-dose exposure during early life on behavioral development in children. Associations varied between tertiles, underlining the possible non-linear relationships between PFAS exposure and neurobehavioral outcomes. Finally, the point estimates might suggest different associations in girls and boys, emphasizing the possibility that mechanisms through which PFAS exposure exerts effects could be sex-specific.

This is the first study in which the association between PFASs in cord plasma and behavioral development in young children has been explored, although studies assessing the association between PFASs in serum of older children or in maternal serum have been conducted. In the current study, negative associations were found between PFOA exposure and externalizing behavior in boys, and between ΣPFAS exposure and the same outcome in girls. In previous studies, some authors reported positive associations between PFAS levels in bodily fluids and behavioral outcomes in children [42,43]. However, results do not seem to be consistent as others reported both positive and negative associations, depending on the compound and type of analysis (crude/adjusted) [41,63], and yet others reported no associations between PFAS exposure and behavioral development at all [64,65,66,67]. When reviewing studies reporting negative associations, increased childhood PFOA exposure has been related to a decrease in attention and ADHD characteristics in school-aged children [41,68]. In another study on the same cohort, PFOA exposure in boys aged 6 to 12 years was negatively associated with ADHD [25]. In literature, using maternal reports on child behavior, negative associations for boys were found for the association between PFOA exposure and executive functions, externalizing behavior and adaptive skills [25]. This seems to correspond with the results from the current study, although exposure levels in the former studies were higher than in our study and the participants were older [25,69]. 

The PFASs have strong chemical similarities; therefore, these compounds may share a common pathway towards the exhibition of an adverse outcome such as specific behavioral disorders in children, referred to as an adverse outcome pathway (AOP). An AOP provides a conceptual construct to organize the knowledge on an association between a molecular initiating event and an adverse outcome at the biological level [70]. Therefore, AOPs are typically a series of sequential events across several levels of biological organization.

In a recent study, Power *et al*. suggested that exposure to PFOA may have neuroprotective effects, as negative associations were found between exposure to PFASs and cognition in older human adults [25,69]. The authors suggest that PFASs activate the peroxisome proliferator-activated receptor (PPAR) alpha and, to a lesser degree, gamma. Hence, PFASs function as an agonist of these receptors [25,71]. *In vitro* studies have shown that agonists of PPAR alpha and gamma have both neuroprotective as well as central nervous system anti-inflammatory characteristics [25,72,73,74]. Previous *in vitro* work as well as human studies have shown that exposure to PFASs suppresses the immune function [75,76,77]. As neurodegenerative diseases are characterized by inflammation, exposure to PFASs may have a beneficial impact on brain health in older human adults [69]. Indeed, PPAR agonists are used for the treatment of certain neurodegenerative diseases, and may protect against cognitive impairment [69]. These results have not yet been demonstrated in children in relation to ADHD, but it has been proposed that children might also benefit from these neuroprotective properties, by means of improved executive functioning and attention skills [25].

In this study, we found some evidence for sex-specific associations, even though interactions between exposure and sex were not statistically significant. However, previous studies reported sex differences for early-life exposure to PFASs and child health [49,78], underlining the idea that early life exposure to environmental toxicants is associated with different health effects for males and females [79,80]. The mechanisms of action which underlie these sex-specific associations are not fully understood, but prenatal PFOA exposure has been related to changes in gonadal hormone levels, such as reduced testosterone levels, and increased estradiol levels [81,82]. Susceptibility to PFASs might be explained by sex differences in these prenatal gonadal hormone levels [80,83,84].

The strengths of this study include the use of biological sampling to determine levels of exposure to PFASs, which means exposure could be determined prospectively by making use of objective instruments. In addition, the CBCL is a well-known and widely used screening instrument for behavioral problems, which increases comparability with other studies. The CBCL 1.5–5 has well-established reliability and validity, is standardized in many countries and has been translated into nearly 60 languages [85]. Therefore, the CBCL is a useful primary screening method for psychopathology, which should however be combined as a screening tool with in-depth assessment tools such as clinical observation for diagnosis purposes. Another strength of the study includes the rather homogeneous study group, which makes confounding by socioeconomic or demographic factors less likely. 

Limitations of the current study include the small sample size, which has reduced the power of the statistical tests, especially tests for interaction and those in the sex-stratified analyses. In addition, there is also a large probability for type I errors, given the many comparisons that were tested. We chose not to adjust our significance levels, because that would further reduce the statistical power of our tests. In some previous studies, higher serum levels of PFOA were related to higher educational levels [86,87,88]. Therefore, it has been suggested that levels of PFASs could be a surrogate marker of educational level. However, when exploring the correlations between PFASs and maternal educational levels, no significant associations could be found in the current study. However, it should be emphasized that, as a result, our findings should be seen as exploratory and in need of corroboration from future larger-scale studies. Finally, some of the covariates, such as alcohol or illicit drug use during pregnancy, were measured using self-reports (yes/no), which may reduce reliability and validity of these data.

## 5. Conclusions 

In conclusion, this is one of the first studies in which prenatal PFAS exposure has been related to behavioral development in preschool children. Prenatal exposure to increasing levels of PFOA was related to less externalizing behavior in boys. A negative association was found between ΣPFAS exposure and externalizing problem behavior in the whole study population and, after sex-stratification, in girls. Results should be interpreted with caution, as the sample size was small. 

Although the current study seems to indicate protective effects of exposure to PFASs, previous reported adverse health effects should be taken into account. Considering the burden developmental disorders place on both the medical and educational system, it should be a priority to identify risk factors that might be modifiable. As knowledge on the relationship between exposure to PFASs and behavioral disorders later in life is limited, more research in this area is also needed.

## Figures and Tables

**Table 1:** Exposure levels of the various endocrine-disrupting chemicals in cord plasma, expressed in ng/L as well as nM.

Compound	*N*	Unit of Conc.	Mean (SD)	Median	Range	LOQ	<LOQ (%)
PFOA	59	ng/L	905.6 (437.1)	870.0	200–2300	50–140	0
	59	nM	2.19 (1.06)	2.10	0.48–5.55	0.12–0.34	0
PFOS	59	ng/L	1583.6 (648.3)	1600.0	570–3200	44–140	0
	59	nM	3.17 (1.30)	3.21	1.14–6.41	0.09–0.28	0
PFHxS	59	ng/L	140.0 (69.2)	145.0	36.0–260.0	47–100	1
	59	nM	0.41 (0.19)	0.38	0.06–1.00	0.09–0.25	1
PFHpS	59	ng/L	35.6 (21.3)	32.0	5.10–120.0	3.7–8.2	0
	59	nM	0.08 (0.05)	0.07	0.01–0.27	0.09–0.02	0
PFNA	59	ng/L	140.0 (61.8)	140.0	60.0–440.0	19–42	0
	59	nM	0.32 (0.13)	0.30	0.13–0.95	0.03–0.09	0
PFDA	59	ng/L	52.2 (20.9)	46.0	23.0–130.0	11–28	0
	59	nM	0.10 (0.04)	0.09	0.04–0.25	0.02–0.05	0
PFUnDA	59	ng/L	32.05 (11.9)	27.5	22.0–67.0	130–290	1
	59	nM	0.03 (0.02)	0.02	0.01–0.12	0.03–0.07	1
Sum PFASs	59	ng/L	2907.0 (1051.5)	2907.0	982–5969	165–404	0
	59	nM	6.30 (2.29)	6.24	2.08–13.12	0.37–1.01	0

LOQ = limit of quantification; *N* = sample size; ng/L = nanogram per liter; PFASs = perfluoroalkyl substances; PFOA = perfluorooctanoic acid; PFOS = perfluorooctanesulfonic acid; SD = standard deviation; Conc. = concentration.

**Table 2:** Maternal and CBCL data for the study population.

Characteristic	Whole Sample (*N* = 57–59)		Boys (*N* = 34–38)		Girls (*N* = 20–21)	
		Range		Range		Range
Gestational age in weeks (mean, SD)	40.0 (1.1)	36.6–42.0	39.9 (1.2)	37.2–41.6	40.1 (1.1)	36.6–42.0
Age mother in years (mean, SD)	31.3 (3.8)	23.0–40.0	30.9 (3.8)	24.0–40.0	32.1 (3.6)	23.0–36.0
Educational level (high, %)	43 (74.1)	-	29 (78.4)	-	14 (66.7)	-
Smoking pregnancy (yes, %)	3 (5.3)	-	2 (5.6)	-	1 (4.8)	-
Alcohol use pregnancy (yes, %)	6 (10.3)	-	4 (10.5)	-	2 (10.0)	-
Illicit drug use pregnancy (yes, %)	0 (0)	-	0 (0.0)	-	0 (0.0)	-
Family history ADHD (yes, %)	8 (14.3)	-	6 (16.7)	-	2 (10.0)	-
ADHD score (mean, SD)	3.6 (1.9)	0–8	3.3 (1.8)	0–7	3.6 (1.5)	1–7
Externalizing behavior score (mean, SD)	9.7 (5.7)	1–24	9.2 (6.0)	1–22	9.6 (4.7)	1–19

ADHD = Attention Deficit Hyperactivity Disorder; CBCL = Child Behavior Checklist; *N* = sample size; SD = standard deviation.

## Appendix

**Table A1:** Regression coefficients for exposure to perfluoroalkyl substances and scores on the Attention Deficit Hyperactivity Disorder scale of the CBCL, for the whole population.

Compound	*N*	Matrix	Unit of Conc.	T1	T2		T3	
					β (95% CI)	*p*-Value	β (95% CI)	*p*-Value
PFOA				200.0−640.0	640.1−1000.0		1000.1−2300.0	
*Crude model*	53	CP	ng/L	Ref.	−0.18 (−1.56; 1.11)	0.78	−0.31 (−1.35; 0.79)	0.55
*Adjusted model* ^1^	53	CP	ng/L	Ref.	−0.33 (−1.99; 1.20)	0.70	−0.24 (−1.65; 0.85)	0.72
PFOS				570.0−1200.0	1200.1−1800.0		1800.1−3200.0	
*Crude model*	53	CP	ng/L	Ref.	−0.27 (−1.58; 1.09)	0.70	−0.62 (−1.67; 0.41)	0.26
*Adjusted model* ^2^	53	CP	ng/L	Ref.	−0.33 (−1.75; 1.17)	0.66	−0.87 (−2.06; 0.42)	0.19
Sum PFASs				982.0−2517.8	2517.9−3337.7		3337.8−5969.0	
*Crude model*	53	CP	ng/L	Ref.	−0.79 (−2.06; 0.49)	0.23	−0.81 (−1.82; 0.17)	0.13
*Adjusted model* ^3^	53	CP	ng/L	Ref.	−0.72 (−1.96; 0.59)	0.31	−0.99 (−2.14; 0.18)	0.11
Sum PFASs (nM)				2.08−5.36	5.37−7.21		7.22−13.12	
*Crude model*	59	CP	nM	Ref.	−0.84 (−2.06; 0.43)	0.20	−0.53 (−1.58; 0.58)	0.36
*Adjusted model* ^4^	59	CP	nM	Ref.	−0.84 (−2.05; 0.37)	0.18	−0.53 (−1.62; 0.59)	0.33

β = regression coefficient; BM = breastmilk; CI = confidence interval; CP = cord plasma; *N* = sample size; Conc. = concentration; ng/L = nanogram per liter; PFASs = perfluoroalkyl substances; PFOA = perfluorooctanoic acid; PFOS = perfluorooctanesulfonic acid; pg/g lpd = picogram per gram lipid; T1 = tertile 1; T2 = tertile 2; T3 = tertile 3; Ref. = feference group. ^1^ Adjusted for smoking, family history of ADHD, alcohol use, EDU; ^2^ Adjusted for smoking, family history ADHD, alcohol use; ^3^ adjusted for alcohol use, smoking, family history of ADHD; ^4^ No adjustment for covariates.

**Table A2:** Regression coefficients for exposure to perfluoroalkyl substances and scores on the Externalizing Behavior Scale of the CBCL, for the whole population.

Compound	*N*	Matrix	Unit of Conc.	T1	T2		T3	
					β (95% CI)	*p*-Value	β (95% CI)	*p*-Value
**PFOA**				**200.0−640.0**	**640.1−1000.0**		**1000.1−2300.0**	
*Crude model*	54	CP	ng/L	Ref.	−2.50 (−6.39; 1.28)	0.19	−2.00 (−5.62; 1.46)	0.28
*Adjusted model* ^1^	54	CP	ng/L	Ref.	−3.33 (−7.65; 0.29)	0.12	−2.30 (−6.88; 1.55)	0.31
**PFOS**				**570.0−1200.0**	**1200.1−1800.0**		**1800.1−3200.0**	
*Crude model*	53	CP	ng/L	Ref.	−1.24 (−5.15; 2.79)	0.58	−2.08 (−5.31; 1.22)	0.22
*Adjusted model* ^2^	53	CP	ng/L	Ref.	−1.23 (−5.68; 3.85)	0.62	−2.43 (−6.55; 1.93)	0.31
**Sum PFASs**				**982.0−2517.8**	**2517.9−3337.7**		**3337.8−5969.0**	
*Crude model*	56	CP	ng/L	Ref.	−1.84 (−5.71; 2.04)	0.33	−3.19 (−6.10; −0.07)	0.04
*Adjusted model* ^3^	56	CP	ng/L	Ref.	−1.59 (−5.27; 2.50)	0.45	−3.31 (−6.55; 0.28)	0.07
**Sum PFASs (nM)**				**2.08−5.36**	**5.37−7.21**		**7.22−13.12**	
*Crude model*	58	CP	nM	Ref.	−2.82 (−6.50; 0.95)	0.15	−3.80 (−6.86; −0.79)	0.03
*Adjusted model* ^4^	58	CP	nM	Ref.	−2.83 (−6.40; 0.99)	0.14	−4.11 (−7.49; −0.68)	0.03

β = regression coefficient; BM = breastmilk; CI = confidence interval; CP = cord plasma; *N* = sample size; Conc. = concentration; ng/L = nanogram per liter; PFASs = perfluoroalkyl substances; PFOA = perfluorooctanoic acid; PFOS = perfluorooctanesulfonic acid; pg/g = picogram per gram; T1 = tertile 1; T2 = tertile 2; T3 = tertile 3; Ref. = reference group. ^1^ Adjusted for smoking, family history of ADHD, alcohol use, EDU; ^2^ Adjusted for smoking, family history ADHD, alcohol use; ^3^ adjusted for alcohol use, smoking, family history of ADHD; ^4^ No adjustment for covariates.

**Table A3:** Regression coefficients for exposure to perfluoroalkyl substances and scores on the Attention Deficit Hyperactivity Disorder scale of the CBCL, stratified for sex.

Compound	Boys						Girls					
	*N*	T1	T2		T3		*N*	T1	T2		T3	
			β (95% CI)	*p*-Value	β (95% CI)	*p*-Value			β (95% CI)	*p*-Value	β (95% CI)	*p*-Value
**PFOA**		**200.0****–670.0**	**670.1****–1000.0**		**1000.1****–2300.0**			**300.0****–560.0**	**560.1****–909.9**		**910.0****–1700.0**	
*Crude model*	34	Ref.	−0.64 (−2.40; 1.27)	0.54	−0.83 (−2.34; 0.75)	0.32	19	Ref.	0.38 (−0.97; 1.71)	0.59	0.38 (−1.41; 2.18)	0.68
*Adj. model* ^1^	34	Ref.	−1.13 (−3.13; 0.79)	0.27	−1.06 (−2.82; 0.45)	0.22	19	Ref.	0.31 (−2.44; 3.33)	0.79	0.68 (−0.66; 2.11)	0.31
**PFOS**		**570.0****–1300.0**	**1300.1****–1800.0**		**1800.1****–2900.0**			**570.0****–906.7**	**906.8****–1866.5**		**1866.6****–3200.0**	
*Crude model*	34	Ref.	−0.42 (−2.31; 1.41)	0.62	−0.75 (−2.21; 0.71)	0.32	19	Ref.	−0.13 (−1.50; 1.11)	0.87	−0.48 (−1.97; 1.20)	0.56
*Adj. model* ^2^	34	Ref.	−0.55 (−2.84; 1.57)	0.64	−0.99 (−3.03; 0.92)	0.35	19	Ref.	0.17 (−1.50; 1.67)	0.85	−0.73 (−2.36; 0.90)	0.43
**Sum PFASs**		**982.0****–2665.9**	**2666.0****–3337.8**		**3337.9****–5969.0**			**1121.0****–1894.9**	**1895.0****–3618.2**		**3618.3****–4622.0**	
*Crude model*	34	Ref.	−1.04 (−2.81; 0.72)	0.26	−1.34 (−2.80; 0.10)	0.07	20	Ref.	−0.85 (−2.45; 0.80)	0.33	−0.10 (−1.38; 1.47)	0.92
*Adj. model* ^3^	34	Ref.	−1.06 (−2.82; 0.73)	0.65	−1.33 (−3.00; 0.27)	0.11	20	Ref.	−0.85 (−2.50; 0.78)	0.38	−0.60 (−1.99; 0.83)	0.43
**Sum PFASs (nM)**		**2.08****–5.80**	**5.81****–7.21**		**7.22****–13.12**			**2.44****–4.15**	**4.16****–7.76**		**7.77****–9.99**	
*Crude model*	38	Ref.	−1.04 (−2.53; 0.68)	0.24	−1.21 (−2.67; 0.46)	0.13	20	Ref.	−0.92 (−2.69; 0.56)	0.30	0.33 (−1.30; 2.17)	0.74
*Adj. model* ^4^	38	Ref.	−1.03 (−2.81; 0.73)	0.23	−1.32 (−2.86; 0.43)	0.12	20	Ref.	−1.10 (−2.84; 1.01)	0.30	0.57 (−1.00; 2.55)	0.57

β = regression coefficient; Adj. = adjusted; BM = breastmilk; CI = confidence interval; CP = cord plasma; *N* = sample size; ng/L = nanogram per liter; PFASs = perfluoroalkyl substances; PFOA = perfluorooctanoic acid; PFOS = perfluorooctanesulfonic acid; pg/g lpd = picogram per gram lipid; T1 = tertile 1; T2 = tertile 2; T3 = tertile 3; Ref. = Reference group. ^1^ Adjusted for smoking, family history of ADHD, alcohol use, EDU; ^2^ Adjusted for smoking, family history ADHD, alcohol use; ^3^ adjusted for alcohol use, smoking, family history of ADHD; ^4^ no adjustment for covariates.

**Table A4:** Regression coefficients for exposure to perfluoroalkyl substances and scores on the Externalizing Behavior Scale of the CBCL, stratified for sex.

Compound	Boys						Girls					
	*N*	T1	T2		T3		*N*	T1	T2		T3	
			β (95% CI)	*p*-Value	β (95% CI)	*p*-Value			β (95% CI)	*p*-Value	β (95% CI)	*p*-Value
**PFOA**		**200.0****–670.0**	**670.1****–1000.0**		**1000.1****–2300.0**			**300.0****–560.0**	**560.1****–909.9**		**910.0****–1700.0**	
*Crude model*	34	Ref.	−4.32 (−9.62; 1.42)	0.13	−4.61 (−10.18; 0.59)	0.11	20	Ref.	−0.31 (−6.87; 6.56)	0.93	1.56 (−2.65; 5.55)	0.47
*Adjusted model* ^1^	34	Ref.	−5.87 (−10.76;−0.43)	0.05	−5.54 (−11.57; −0.29)	0.09	20	Ref.	−5.24 (−12.82; 0.00)	0.10	0.71 (−3.83; 5.21)	0.74
**PFOS**		**570.0****–1300.0**	**1300.1****–1800.0**		**1800.1****–2900.0**			**570.0****–906.7**	**906.8****–1866.5**		**1866.6****–3200.0**	
*Crude model*	34	Ref.	0.65 (−4.27; 6.21)	0.80	−0.77 (−5.30; 3.45)	0.74	20	Ref.	−3.55 (−9.22; 2.59)	0.26	−3.89 (−8.57; 0.68)	0.11
*Adjusted model* ^2^	34	Ref.	0.72 (−5.77; 6.59)	0.81	−0.94 (−6.72; 5.12)	0.74	20	Ref.	−2.63 (−8.21; 4.33)	0.44	−2.98 (−8.08; 2.23)	0.31
**Sum PFASs**		**982.0****–2665.9**	**2666.0****–3337.8**		**3337.9****–5969.0**			**1121.0****–1894.9**	**1895.0****–3618.2**		**3618.3****–4622.0**	
*Crude model*	34	Ref.	−0.84 (−6.27; 4.61)	0.77	−4.01 (−9.48; 1.38)	0.16	20	Ref.	−6.30 (−11.42; −0.99)	0.03	−2.30 (−5.65; 1.02)	0.22
*Adj. model* ^3^	34	Ref.	−1.09 (−7.22; 5.95)	0.74	−4.20 (−10.73; 2.08)	0.22	20	Ref.	−9.50 (−14.21; −4.35)	0.01	−3.29 (−7.69; 0.66)	0.13
**Sum PFASs (nM)**		**2.08****–5.80**	**5.81****–7.21**		**7.22****–13.12**			**2.44****–4.15**	**4.16****–7.76**		**7.77****–9.99**	
*Crude model*	38	Ref.	−2.05 (−7.07; 3.11)	0.44	−4.75 (−9.62; 0.13)	0.07	20	Ref.	−6.22 (−11.28; −0.50)	0.03	−1.37 (−5.50; 2.79)	0.49
*Adj. model* ^4^	38	Ref.	−2.04 (−6.77; 3.21)	0.42	−4.93 (−9.56; 0.69)	0.07	20	Ref.	−5.29 (−11.00; 0.67)	0.09	−0.43 (−4.67; 4.47)	0.85

β = regression coefficient; Adj. = adjusted; BM = breastmilk; CI = confidence interval; CP = cord plasma; *N* = sample size; ng/L = nanogram per liter; PFASs = perfluoroalkyl substances; PFOA = perfluorooctanoic acid; PFOS = perfluorooctanesulfonic acid; pg/g lpd = picogram per gram lipid; T1 = tertile 1; T2 = tertile 2; T3 = tertile 3; Ref. = reference group. ^1^ Adjusted for smoking, family history of ADHD, alcohol use, EDU; ^2^ Adjusted for smoking, family history ADHD, alcohol use; ^3^ adjusted for alcohol use, smoking, family history of ADHD; ^4^ No adjustment for covariates.

## Abbreviations

The following abbreviations are used in this manuscript:

ADHD	Attention Deficit Hyperactivity Disorder
CBCL 1.5–5	Child Behavior Checklist 1.5–5
LOQ	Limit of quantification
PFASs	Perfluoroalkyl substances
PFDA	Perfluoro-n-decanoic
PFHxS	Perfluorohexane sulfonate
PFHpS	Perfluoroheptane sulfonate
PFNA	Perfluorononanoic acid
PFOA	Perfluorooctanoic acid
PFOS	Perfluorooctanesulfonic acid
PFUnDA	Perfluoroundecanoic acid
